# Adaptation of H9N2 Influenza Viruses to Mammalian Hosts: A Review of Molecular Markers

**DOI:** 10.3390/v12050541

**Published:** 2020-05-14

**Authors:** Xiangjie Sun, Jessica A. Belser, Taronna R. Maines

**Affiliations:** Influenza Division, National Center for Immunization and Respiratory Diseases, Centers for Disease Control and Prevention, Atlanta, GA 30333, USA; jax6@cdc.gov (J.A.B.); zay9@cdc.gov (T.R.M.)

**Keywords:** Influenza A virus, mammalian host adaptation, molecular markers

## Abstract

As the number of human infections with avian and swine influenza viruses continues to rise, the pandemic risk posed by zoonotic influenza viruses cannot be underestimated. Implementation of global pandemic preparedness efforts has largely focused on H5 and H7 avian influenza viruses; however, the pandemic threat posed by other subtypes of avian influenza viruses, especially the H9 subtype, should not be overlooked. In this review, we summarize the literature pertaining to the emergence, prevalence and risk assessment of H9N2 viruses, and add new molecular analyses of key mammalian adaptation markers in the hemagglutinin and polymerase proteins. Available evidence has demonstrated that H9N2 viruses within the Eurasian lineage continue to evolve, leading to the emergence of viruses with an enhanced receptor binding preference for human-like receptors and heightened polymerase activity in mammalian cells. Furthermore, the increased prevalence of certain mammalian adaptation markers and the enhanced transmissibility of selected viruses in mammalian animal models add to the pandemic risk posed by this virus subtype. Continued surveillance of zoonotic H9N2 influenza viruses, inclusive of close genetic monitoring and phenotypic characterization in animal models, should be included in our pandemic preparedness efforts.

## 1. Introduction

Genetically diverse influenza A viruses can be grouped by their surface glycoproteins into 18 hemagglutinin (HA) subtypes and 11 neuraminidase (NA) subtypes [1]. Influenza A viruses possess a wide host range, and almost all subtypes (with the exception of H17N10 and H18N11 viruses) have been identified in wild aquatic birds, which are regarded as the natural reservoir for influenza A viruses [2,3]. However, only a subset of these virus subtypes has successfully established documented mammalian infection. Host barriers largely restrict virus transmission between different species, but when these barriers are breached, cross-species transmission of influenza viruses may occur and cause severe consequences, especially during virus transmission from avian hosts to mammals [4,5]. In humans, H1, H2 and H3 viruses with select gene segments or entire genomes derived from influenza viruses adapted to avian or swine hosts have caused pandemics during the 20th and 21st centuries, resulting in profound effects on public health. Meanwhile, additional influenza A subtypes of avian origin, including H5, H6, H7, H9 and H10, have caused sporadic human infection, with H5 and H7 avian influenza viruses responsible for the majority of human cases [4,6]. A number of comprehensive risk assessment studies of H5 and H7 subtype avian influenza viruses have been reported in recent years [7,8,9]. In contrast, less attention has been paid to the pandemic potential of H9 subtype influenza A viruses, particularly H9N2 viruses. In this review, we focus on a risk assessment of H9N2 viruses, with a particular emphasis on key molecular markers associated with host adaptation that are present among contemporary H9N2 viruses. 

## 2. Risk Assessment of H9N2 Influenza Viruses

### 2.1. Emergence and Host Range of H9N2 Viruses

Avian influenza viruses of the H9N2 subtype were first reported in North American turkey flocks in 1966 [10]. Currently, avian influenza viruses of this subtype continue to be detected in North America at a very low frequency among wild birds and turkeys, but not in chickens [11,12]. Worldwide detection of H9N2 viruses in land-based poultry was first reported in the 1990s [13]. Since this time, H9N2 viruses have become endemic in domestic poultry in Asia, the Middle East and Northern Africa [14,15], and represent the most prevalent influenza virus subtype in poultry detected in China during the past 25 years [16]. Despite possessing a low pathogenicity phenotype in chickens, H9N2 virus infection can cause significant economic losses to poultry industries, especially during coinfection with other pathogens. Concurrent with H9N2 virus circulation in domestic poultry, these viruses have also caused documented infection in numerous mammalian species, including pigs, minks, horses, canines, and humans [17,18,19]. To date, H9N2 viral infection in pigs has been primarily limited to China, with a number of outbreaks in swine herds reported since their first detection in this species in Hong Kong in 1989 [20]. 

The first documented human infections with a H9N2 virus occurred in Hong Kong in 1999. To date, over 60 confirmed human cases, including one fatal case, have been reported to the WHO [21,22]; the majority of infected persons have presented with mild respiratory symptoms. However, retrospective human serological studies examining H9N2 exposure have revealed that 2.3%–15% of workers exposed to poultry in China have detectable antibodies against the H9 influenza virus HA protein [23,24,25,26], with even higher rates of seropositivity (30%–85%) reported among poultry workers in Pakistan [27]. The extended tropism of H9N2 viruses in mammals has highlighted the capacity for cross-species transmission of this virus, illustrating that close monitoring of virus evolution of this subtype in various hosts is essential for pandemic preparedness. 

### 2.2. Genetic Diversity and Reassortment of H9N2 Viruses

H9N2 viruses have continued to evolve and reassort with other virus subtypes in avian hosts since their first detection in 1966, leading to substantial genetic diversity [28,29]. The viruses can be generally grouped into two major lineages (North American and Eurasian) based on HA phylogenetic analyses. Viruses in the Eurasian lineage have further evolved into three distinct clusters: the A/quail/Hong Kong/G1/1997 (G1) lineage, which is most prevalent in Northern Africa, the Middle East and southern China; the A/chicken/Beijing/1/1994 (Y280/G9) lineage, which is prevalent in China and Viet Nam [30]; and the A/duck/Hong Kong/Y439/1997 (Y439-like) lineage, which is primarily found in chickens in Korea [31]. Both G1 and Y280/G9 lineages exhibit substantial genetic diversity; the Y280/G9 lineage has been further grouped into A and B sublineages (Belser, et al. manuscript in submission). To date, only H9N2 viruses from the G1 and Y280/G9 lineages have been reported to cause human infection [26]. The widespread distribution of influenza viruses in poultry throughout China has led to extensive virus reassortment and generation of diverse genotypes [32]. Meanwhile, the internal genes of H9N2 viruses have exhibited high compatibility with surface genes from other avian influenza virus subtypes. For example, H5N1, H5N6, H7N9 and H10N8 viruses, which have caused human infection, have all acquired internal genes from H9N2 viruses [33,34,35,36,37]. 

### 2.3. Pathogenesis and Transmission of H9N2 Viruses in Mammals

Numerous H9N2 viruses, representative of different host species but mainly limited to the G1 and Y280/G9 lineages, have been evaluated for pathogenicity and transmissibility in various mammalian animal models. To cause a pandemic, an antigenically novel influenza virus needs to be capable of causing disease and transmitting in a sustained manner among people. Influenza virus infection in both mouse and ferret models provides valuable information towards mammalian disease and adaptation, with data from ferret and guinea pig models providing additional information on virus transmission in mammalian hosts. H9N2 viruses typically cause infection in mice without prior adaptation, although virulence varies from asymptomatic infection to lethal outcome depending on the virus strain [38,39]. Serial passage of H9N2 viruses in this species has led to acquisition of molecular determinants of virulence that can result in enhanced disease in this model [40]. However, due to the genetic diversity of H9N2 viruses, a complete understanding of viral gene segments or key residues determinative to pathogenesis in the mouse model has not been realized.

Guinea pigs do not exhibit pronounced clinical signs following infection with influenza A viruses, and are not routinely used for viral pathogenesis studies [41]. However, H9N2 virus transmission has been studied in guinea pigs, as this species supports transmission of human and some zoonotic influenza viruses [42]. Wild-type H9N2 viruses have exhibited various levels of transmission in guinea pig in the presence of direct contact, but do not transmit efficiently via the airborne route [40,43,44]. However, serial passage of a H9N2 virus in guinea pigs resulted in a virus that displayed efficient transmission in a direct contact model, attributable to mutations in the HA and NP [45]. A separate study identified that inclusion of a PA gene from a 2009 pandemic H1N1 (pdm09H1N1) virus on the backbone of an avian H9N2 virus was sufficient to confer transmission in a direct contact model [46], which revealed multiple paths for H9N2 viruses to acquire enhanced transmissibility in mammals.

In ferrets, H9N2 viral infections generally cause mild disease; virus replication is typically limited to the upper respiratory tract, with occasional spread to the lower respiratory tract [39]. However, the transmissibility of H9N2 viruses in the ferret model varies, depending on the genetic background of the particular strain. Examination of a panel of six H9N2 viruses isolated between 1978 and 2011, inclusive of different host species, clades, and geographical regions, showed that, despite comparable viral titers in ferret nasal wash specimens, only one human isolate (A/Hong Kong/33982/2009) and one avian isolate (A/chicken/Hong Kong/G9/1997) were capable of efficient transmission in a direct contact (but not respiratory droplet) setting [39]. Similarly, using a group of H9N2 viruses isolated from different avian species in China and the Middle East from 1988 to 2003, Wan et al. identified two H9N2 viruses (A/Guinea fowl/Hong Kong/WF10/1999 and A/Duck/Hong Kong/Y280/1997), which exhibited the ability to transmit between ferrets in a direct contact model [47]. Later, Li et al. characterized 35 H9N2 viruses isolated from poultry in China between 2009 and 2013, and found that 6 of them that were representative of the different genotypes circulating in China exhibited the ability to replicate and transmit by respiratory droplets in ferrets [38]. Most recently, Belser et al. assessed the pathogenicity and transmissibility of three contemporary H9N2 human isolates in the ferret model and revealed that A/Anhui-Lujiang/39/2018 virus displayed an increased capacity for replication and respiratory droplet transmission compared to two early human isolates (A/Hong Kong/1073/1999 and A/Hong Kong/308/2014) (Belser et al., manuscript in submission). 

The transmissibility of H9N2 viruses in mammalian hosts has been further studied with the aid of reverse genetics approaches, in which recombinant viruses with the HA and NA genes from a H9N2 virus and the internal genes from a human seasonal influenza virus are generated and assessed for transmission between ferrets. Wan et al. rescued a reassortant virus bearing the HA and NA genes from A/Guinea fowl/Hong Kong/WF10/1999 (H9N2) and the internal genes from a seasonal H3N2 virus, and found that the reassortant virus was not capable of airborne transmission unless the virus was adapted in ferrets through multiple passages [48]. Interestingly, a reassortant virus with the HA and NA from the same H9N2 virus and internal genes derived from a pdm09H1N1 virus exhibited limited airborne transmission without ferret adaptation [49], suggesting that the surface proteins from H9N2 viruses might already possess the minimum requirements for transmission through the air when internal genes provide adequate viral replication capacity. The capability for reassortment between H9N2 and seasonal influenza viruses and the enhanced airborne transmission exhibited by certain contemporary H9N2 viruses underscores the pandemic risk posed by this virus subtype and necessitates the identification of causative molecular signatures in viral genes.

## 3. Key Molecular Markers in H9N2 Influenza Viruses Associated with Host Adaptation

A better understanding of the molecular requirements for influenza virus adaptation to mammalian hosts improves surveillance efforts and pandemic risk assessments of zoonotic influenza viruses, including but not limited to those within the H9 subtype. The HA and polymerase genes, which have been shown to play important roles in virus host range and adaptation [5,50], are natural targets for genetic analyses. Below, we will focus on selected mammalian adaptation markers among H9N2 viruses and evaluate the potential public health risk posed by viruses bearing these markers. 

### 3.1. Hemagglutinin Protein

#### 3.1.1. Receptor Binding

As one of the two types of glycoproteins on the viral surface, the influenza virus HA plays several important functions during the influenza virus life cycle, including critical roles in receptor-mediated binding to host epithelial cells and subsequent fusion between the viral envelope and host endosomal membranes [51]. Influenza viruses of avian and human origin have evolved to possess distinct receptor binding specificities, with human-origin viruses preferentially binding to α2,6-linked sialic acid moieties and avian-origin viruses preferentially binding to those with α2,3-linked sialic acid [52]. Experimental studies supported by HA crystallographic structural analyses have demonstrated that influenza virus receptor binding specificity is governed by a few key residues located in the HA head region. For H2 and H3 viruses, the key residues for receptor binding specificity are amino acids 226 and 228 (H3 numbering used throughout the text), with the presence of leucine (L) at 226 and serine (S) at 228 favoring virus binding to human-like receptors and the presence of glutamine (Q) at 226 and glycine (G) at 228 favoring virus binding to avian-like receptors [53]. H9N2 viruses have exhibited variation at position 226, with the majority of viruses possessing either a Q or L at this residue, whereas G228 is conserved among almost all H9 viruses (Table 1).

To further understand H9N2 virus receptor binding specificity from a genetic perspective, we performed an analysis of full-length HA sequences from more than 2500 avian isolates as well as the H9N2 isolates from mammalian hosts deposited in GISAID (Table 1), which revealed a shift in the predominant residue at position 226 during virus evolution in avian hosts. The majority (85%) of H9N2 viruses isolated from birds prior to 1999 possess Q226, indicating an avian-like receptor binding preference. In contrast, contemporary H9N2 avian isolates exhibit a higher prevalence of L226, with 72% of viruses from 1999 to 2012 and 95% of viruses from 2013 to 2019, suggesting an increased capacity for human-like receptor binding. With regard to H9N2 viruses isolated from mammalian hosts, 29 out of 34 (85.7%) human isolates and 20 out of 35 (57.1%) isolates from nonhuman mammals with full-length HA sequences available in GISAID contain L226 and G228. 

The receptor binding specificity and affinity of H9N2 viruses has also been experimentally evaluated in vitro by many groups [31,54,55,56]. However, despite the dominant role in receptor binding played by residue 226 for H2 and H3 viruses, the correlation between HA L226 and virus binding to human-like receptors varies substantially depending on the methods utilized to assess receptor binding and the particular virus strains being tested. Some studies show H9N2 viruses bearing HA L226 preferentially bind to human-like receptors [38,45,54,55,57], whereas others show negligible binding to human-like receptors [31] [Belser, et al. manuscript in submission]. This variation in H9N2 virus binding data suggests that influenza virus receptor binding is a complex property and can be influenced by the nature and the combination of multiple residues in the HA as well as other factors such as NA stalk length and sialidase activity. The role of HA L226 in H9N2 virus receptor binding has also been studied in relevant cell types, tissues, and animal transmission models. Wan et al. demonstrated that H9N2 viruses bearing HA L226 exhibited a binding preference for nonciliated cells, similar to human seasonal influenza viruses, and displayed a higher replicative capacity in human airway epithelial cells compared to H9N2 viruses possessing Q226 [58]. Additionally, the presence of HA L226 in the H9N2 virus, A/Guinea fowl/Hong Kong/WF10/1999, was associated with both efficient viral replication in the ferret upper respiratory tract and enhanced transmission in the presence of direct contact, but not airborne, ferret transmission model. Importantly, introduction of a single L226Q substitution in the HA of this virus reduced viral replication and abolished transmission [47]. 

Beyond HA positions 226 and 228, additional residues previously identified to contribute to virus receptor binding specificity and affinity of H1 and H3 viruses, notably those located at 155, 183, and 190 [59], have been studied on the genetic background of H9N2 viruses [54,55]. The I155T substitution in select H9 viruses led to enhanced human-like receptor binding in the absence of L226 [38]. Our genetic analysis revealed that the frequency of I155T in H9 isolates has remained consistently high (>95%) since their emergence, indicating an intrinsic ability for binding to human-like receptors among H9N2 viruses. The role of the H183N substitution in enhancing human-like receptor binding has been primarily studied in H3 viruses [60]. Interestingly, an N183 substitution has steadily gained predominance among H9N2 viruses, increasing from 34.4% prior to 1999 to 73.2% in virus isolates from 2013 to 2019; most human isolates (26/34 or 76.5%) possess this substitution. Although a single substitution of N183H in the HA of A/chicken/Guangxi/9/1999 virus with a Q226 residue showed a similar binding profile to the wild-type virus in a solid-phase receptor binding assay [38], the role of this residue in virus replication and transmission in vivo requires further study. 

The presence of valine (V) at 190 of the HA, previously shown to modulate receptor binding preference among H1 viruses [59], was similarly found to enhance H9N2 receptor binding affinity for human-like receptors as well as augment murine virulence [61]. Additionally, a group of H9N2 viruses with a aspartic acid (D) or glutamic acid (E) substitution at position 190 exhibited enhanced binding for human-like receptors compared to viruses with an alanine (A) or isoleucine (I) at this position, regardless of the residue at position 226 [31]. In support of this, G1 lineage H9N2 viruses isolated in Pakistan in the period 2014–2016 preferentially bound to a sulfated version of an avian-like receptor analog despite the presence of L226 in the HA; A to a threonine (T) or V at position 190 led to enhanced binding affinity to both avian- and human-like receptors, albeit more pronounced for avian-like receptors [62]. The prevalence of 190T/V has steadily increased among avian H9N2 viruses, rising from 22.9% prior to 1999 to 57.2% from 2013 to 2019; human H9N2 isolates exhibit no substantial differences regarding prevalence at this position compared to contemporary avian H9N2 viruses.

A role for receptor binding for HA position 227 was identified following adaptation of a H9N2 virus to guinea pigs [45]. Furthermore, the combination of acidic residues (D/E) at 190 and a Q at position 227 appears to be associated with human-like receptor binding [31]. However, Q227 has exhibited reduced prevalence among avian and human isolates in recent years, dropping from above 71% before 2013 to 6.5% between 2013 and 2019. Taken together, the available evidence suggests that certain contemporary H9N2 viruses have gained enhanced receptor binding specificity toward human-like receptors, partially due to the increased prevalence of HA L226, with contributions from additional residues, such as 155, 183, 190 and 227. However, compared to human seasonal H3N2 viruses with HA L226/S228, H9N2 viruses with L226/G228 may not yet possess optimum binding for human-like receptors, suggesting that further adaptation would improve replication and spread among humans.

#### 3.1.2. H9N2 Virus Stability and HA Activation

In addition to the aforementioned key residues governing receptor binding specificity and affinity, HA acid stability and the resulting thermostability have also been shown to contribute to mammalian host adaptation and airborne transmission in mammalian hosts [63,64,65]. The HA undergoes irreversible conformational changes upon encountering the low pH environment within host endosomal compartments, which then mediates fusion between viral and host endosomal membranes. Preexposure to a low pH environment without a target membrane will lead to virus inactivation. It has been hypothesized that acid-stable viruses may better maintain infectivity in respiratory droplets or during virus passage through the mildly acidic environment of the mammalian nasal cavity [64]. Most avian influenza viruses, including highly pathogenic H5N1 and low pathogenic H7N9 viruses associated with human infection, fuse at a relatively high pH (5.5–5.8) for HA activation, whereas human influenza viruses are generally activated at a lower pH (5.0–5.5) [63,64]. A reduction in the pH requirement for HA activation has been associated with enhanced airborne transmission of H5 viruses in the ferret model [66,67]. In contrast with most H5 and H7 viruses, a recent study showed that many H9N2 viruses, including both G1 and Y280 lineages, exhibit a relatively low threshold pH for fusion (5.4–5.8) [31]. Selected H9N2 viruses responsible for recent human infections were found to have a pH of fusion between 5.4 and 5.5 [Belser et al., manuscript in submission], which is consistent with another study that reports that some currently circulating H9N2 viruses possess a threshold pH for fusion that falls between the relatively high threshold pH for avian H5 and H7 viruses and the low threshold pH for human-adapted pandemic influenza viruses [31]. Additionally, a mutation identified following H9N2 virus adaptation in guinea pigs (HA2-D46E, HA-375, H3 numbering) enhanced transmission using a direct contact model and was found to modulate receptor binding properties and enhance virus thermostability [45]. Although HA2-D46E has not emerged in natural isolates, N46 has been detected; whether an N at this position modulates pH for fusion or transmission warrants further investigation. Interestingly, one study investigating an avian H9N2 virus identified an essential residue HA-363K for virus airborne transmission between chickens, which has been linked to enhance virus acid stability, suggesting that acid stabilizing residues can emerge from avian hosts and might be able to play a similar role in viral mammalian host transmission [68]. Collectively, future studies identifying the key residues responsible for H9 virus acid stability and investigating their potential effect on virus cross-species transmission will be of great importance for risk assessment.

Overall, H9N2 viruses have exhibited a higher propensity for human receptor binding compared to most avian influenza viruses, attributed primarily, but not exclusively, to the prevalence of a HA L226 residue present among contemporary strains. This is the case for both viruses isolated from the avian reservoir, as well as those that have successfully jumped species barriers to cause self-limiting human infection. Beyond receptor binding preference, the acid-stable H9 HA may further enhance the ability of these viruses to adapt and transmit through the air. Taken together, these data provide compelling support that the pandemic risk of H9 viruses cannot be underestimated.

### 3.2. The H9N2 Polymerase Genes (PB2, PB1, PA) 

The influenza virus polymerase complex is composed of three subunits—polymerase basic proteins 2 (PB2) and 1 (PB1), and polymerase acidic protein (PA)—which are collectively responsible for viral gene transcription and replication with the involvement of host factors. Influenza polymerase genes have been recognized as critical determinants in virus host range restriction and species specificity [69]. A number of residues in the PB2, PB1 and PA have been identified to either enhance avian influenza virus polymerase activity in mammalian cells or contribute to avian influenza virus virulence and transmission in mammalian animal models, pointing to their roles in host adaptation. Although selected polymerase amino acid substitutions have been studied on the genetic background of H9N2 viruses, there is a need for further investigation of some less common mutations in H9N2 viruses that may nonetheless play a role in host range and adaptation (Table 1) [69]. 

#### 3.2.1. Mammalian Adaptation Mutations in PB2 

The influenza PB2 protein is comprised of an N-terminal PB1 binding site and a C-terminal host protein interaction domain. A number of key mutations, especially in the host protein interaction domain, have been identified to be involved in host adaptation [70]. Among these, the PB2-E627K substitution is the most well-studied host adaptation markers among all viral polymerase genes. The presence of an E at this position in almost all avian isolates or a lysine (K) in most human-isolated influenza viruses contributes to optimum interaction between PB2 and host factors from different species [71]. The PB2-E627K mutation was present in the 1918, 1957 and 1968 pandemic viruses [72] and has been identified in a large number of H5N1 and H7N9 human isolates [73,74]. Among H9N2 viruses for which public sequences are available, the frequency of PB2-E627K in avian isolates remains low (1.1% among those reported in GISAID). That said, H9N2 viruses isolated from mammalian hosts (including human, swine, and mink) have exhibited a slightly higher frequency of E627K (3.6%), indicating that H9N2 viruses isolated from mammalian hosts have undergone some degree of adaptation, likely including the capacity for enhanced virus replication in mammals. Among the 18 H9N2 isolates bearing PB2-627K, 10 of them possess HA-226L and 7 of them have HA-226L, 183N and 190A/T, suggesting that these viruses may have an even higher capacity for mammalian adaption. Interestingly, a number of H9 avian isolates (7.4%) and up to 21% of human isolates, possess 627V, shown previously to function as an intermediate between 627E and 627K on the H7N9 genetic background [75]. However, whether the emergence of PB2-627V among avian H9N2 viruses contributes to virus transmissibility in mammals remains unanswered. 

The polymorphism of PB2 at position 590/591 was first identified among pdm09H1N1 viruses containing PB2-E627. It was found that PB2-G590S/Q591R shares a similar structural location and function to the residue PB2-627, and can contribute to virulence and transmission in mammalian models [76,77,78]. Although the presence of the double mutation 590S/591R is extremely rare among H9N2 viruses, our analysis revealed that 590S is present in approximately 10% of avian isolates; the prevalence of 590S is even higher among H9N2 human isolates, with about 30% frequency. Although PB2-590S seems to be less critical for enhanced polymerase activity in mammalian cells compared to 591R, as demonstrated with H5N1 viruses, analysis of X-ray crystal structures has revealed that PB2-590S may help to shield the negative charge from 627E [78]. Nevertheless, the role of 590S in the context of a H9N2 background needs further investigation. 

An asparagine (N) at position PB2-701 was identified during serial passage of an avian H7 virus in mice as a contributor to enhanced replication [79]. Subsequent studies found that the presence of PB2-701N could compensate for the absence of 627K and enhance avian H5N1 and pdm09H1N1 virus replication, as well as confer increased transmissibility in mammalian models [80,81,82]. However, only 3 H9 viruses for which public sequences are available possess 701N in their PB2 protein. Additionally, an adjacent residue, PB2-702, has been found to play a role in species specificity, with a prevalence of K among avian influenza viruses and arginine (R) among human influenza viruses; accordingly, H5N1 human isolates have a higher frequency of 702R compared to avian H5N1 viruses [83]. Compared to 701N, the frequency of the mammalian adaptation residue, 702R, among H9 viruses is higher, with 7.5% of avian isolates, and 15.4% and 42.9% of human and nonhuman mammals, respectively.

The role of residue PB2-588 in host adaptation has been demonstrated with pdm09H1N1 virus and different subtypes of avian influenza viruses [84,85]. The substitution PB2-A588V on the H10N8, H7N9, and H9N2 genetic backgrounds results in higher polymerase activity, viral replication, and virulence in mice. Although the molecular mechanism remains unknown, these findings indicate that this residue may contribute to the adaptation of avian influenza viruses to mammalian hosts [86]. Of note, the A588V substitution has shown increased predominance among fifth-wave H7N9 viruses [87]. The proportion of PB2-588V is more dominant among H9N2 viruses compared with other PB2 mammalian adaptation markers, ranging from 17.8% among avian isolates and up to 46.2% among human isolates. Again, whether this residue is involved in H9N2 virus transmission in mammals requires further study.

Numerous additional molecular markers among H9N2 viruses with the potential to affect host adaptation have yet to be fully identified and characterized but will nonetheless warrant increased attention should their prevalence increase. For example, the PB2-K526R substitution was previously shown to enhance viral replication and virulence in mice, particularly in combination with PB2-627K [88]; furthermore, the PB2-K526R substitution has been observed among a number of H7N9 and H5N1 human isolates. H9N2 viruses (both avian and human isolates) currently possess a low frequency (<4%) of PB2-K526R. Additionally, the PB2-T271A substitution was previously found to enhance replication of pdm09H1N1 and H5N1 viruses in mammalian cells in vitro [89,90,91]. This mutation has only been found in 1 of 1472 H9 avian isolates and in 3 of 28 H9 human isolates. Other PB2 amino acids for which a role in H5N1 virus murine virulence has been identified, such as 147T and 339T [83], have not been detected among H9N2 viruses to date.

#### 3.2.2. Mammalian Adaptation Mutations in PB1 and PA 

The influenza virus PB1 protein is predicted to have a characteristic RNA-dependent RNA polymerase structure [70]. The number of identified mammalian adaption mutations in the PB1 protein is relatively limited compared to PB2 and PA. Two human influenza virus signature residues, including PB1-327K and 336I (identified by large-scale genomic analysis) [92], and PB1-H99Y (a substitution identified during H5N1 virus adaptation to ferrets) [66], have not been identified frequently among H9N2 viruses. However, PB1-I368V, which was present in a ferret adapted H5N1 virus [66], has shown increased predominance among H9N2 viruses, with its frequency increasing from 2.8% prior to 1999, to 21% in the period 1999–2012, and to 67% in the period 2013–2019. 

The N-terminus and C-terminus of the PA protein are involved in endonuclease activity and PB1 binding, respectively, and roles for multiple mutations located in these areas have been identified in mammalian host adaptation [70,93,94]. Importantly, contemporary H9N2 viruses have exhibited an increased frequency of substitutions in the PA terminal sequences. PA-356R, located in a short loop region in the C-terminal domain, has been shown to affect viral polymerase activity and virulence in mammals [95,96]. The presence of the K356R mutation in the PA protein of a H9N2 virus resulted in increased viral replication and pathogenicity in mice, either independently or cooperatively with the presence of PB2-627K [97]. The frequency of PA-356R is low (approximately 5%) among sequenced H9N2 isolates prior to 2013. However, its predominance has substantially increased to 82.7% among avian isolates collected in the period 2013–2019; half of human H9N2 isolates also possess this substitution. There is a need to further investigate potential roles for PA-356R in virus transmission employing mammalian models. 

Both PA-100A and 409N have been recognized as characteristic of human influenza virus residues based on computational analyses, as PA-409N exhibited a higher frequency among avian H5N1 viruses isolated from humans compared with avian isolates [83,92]. Analysis of the H9N2 virus PA protein showed that the prevalence of PA-100A and 409N has increased to 15% from less than 1%, and to 83% from 37%, respectively, since 2013. However, none of the H9N2 human isolates with publicly available sequences bears a PA-100A substitution, whereas 60% of them have an N at PA-409. Additional mammalian host adaptation markers, including 85I, 86S, and 336M, which have been demonstrated to enhance polymerase activity in mammalian cells for pdm09H1N1 viruses [98], remain absent or rare in H9N2 viruses.

Taken together, although most H9N2 viruses do not possess well-known mammalian adaptation markers, such as PB2 627K and 701N, the increased detection of amino acid residues, such as PB2-588V, PB1-368V, PA-356R, and PA-409N, in key areas of the H9N2 virus polymerase complex, especially among human isolates, suggests that these viruses continue to adapt to mammalian hosts. Future studies to examine whether such residues can enhance H9N2 virus replication and transmission in mammalian models should be included in our efforts to assess the pandemic potential of emerging H9N2 viruses.

## 4. Conclusions

Influenza pandemics occur when an antigenically novel influenza virus acquires the ability to transmit in a sustained manner among humans. Currently, most avian influenza viruses do not pose an imminent pandemic threat as they cannot efficiently infect and transmit among humans. However, researchers have independently demonstrated that H5N1 viruses, which in wild-type form, do not transmit efficiently through the air in ferret models, can become airborne transmissible when the virus acquires certain mutations conferring an ability to bind to human receptors, display a lower threshold pH for fusion, and replicate efficiently in mammalian cells. This suggests that avian influenza viruses have the potential to cause a pandemic if such mutations emerge [66,67]. Currently, although avian H9N2 viruses have caused fewer documented human infections compared to H5 and H7 viruses, continued evolution of these viruses has resulted in phenotypic and genetic characteristics that indicate an enhanced ability to cause disease and transmit in the human population.

Mammalian pathogenicity, transmissibility, and tropism represent polygenic, multifactorial traits. Roles for all eight gene segments of influenza viruses have been implicated in these properties. However, decades of intense surveillance and pandemic preparedness efforts have supported critical roles for the virus hemagglutinin and polymerase in conferring properties associated with increased host range and adaptation to mammalian species. Here, we have provided an in-depth look at these key proteins of influenza H9N2 viruses and the potential role specific known and suspected molecular determinants of virulence and transmissibility contribute to the relative risk of H9N2 influenza viruses to humans. The hemagglutinin and polymerase proteins represent critical components, but all gene segments of H9N2 viruses are deserving of scrutiny.

Molecular tools have advanced to a point where predictive risk assessment activities may be conducted prior to in vivo experimentation using well-established animal models. Collectively, these evaluations feed into the Tool for Influenza Risk Assessment (TIPRA) and Influenza Risk Assessment Tool (IRAT) [99,100], which better inform infection control strategies and resource allocation decisions. H9N2 viruses have, to date, retained a low pathogenic phenotype in avian species, and have typically been associated with mild, self-limiting human infections. Several key mammalian adaptation markers (including HA-183N,190T/V,227Q, PB2-588V, 627K/V, 701N, PA-356R, and 409N) have emerged among recently isolated H9N2 viruses associated with human infection over the last decade, highlighting the challenge in ensuring that risk assessment rubrics are reflective of contemporary circulating strains. Considering the contribution of selected molecular markers to viral pathogenicity, transmissibility, and host range, there is a need to ensure that these rubrics are inclusive of current pandemic threats and are updated accordingly when warranted. Collectively, their widespread geographic distribution, high propensity for reassortment with other influenza viruses, and exquisitely efficient polymerase complex make H9N2 viruses a key influenza subtype to monitor for pandemic potential. 

## Figures and Tables

**Table 1:** Prevalence of mammalian adaptation markers among H9N2 influenza viruses.

	Location	% of H9N2 Viruses with the Amino Acid Shown					
		<1999	1999–2012	201–2019	Avian Isolates	Human	Nonhuman Mammals
**HA**		(*n* = 96)^c^	(*n* = 1367)	(*n* = 1212)	(*n* = 2675)	(*n* = 34)	(*n* = 35)
	155T (*145*)^a^	100	98.8	99.7	99.2	100	100
	**183N (*173*)^b^**	34.4	64	73.2	67.1	76.5	85.7
	**190T/V (*180*)**	22.9	38.4	57.2	56.3	61.8	60
	**226L (*216*)**	14.6	72.1	95.1	80.4	85.7	57.1
	227Q (217)	97.9	71.4	6.5	43.0	52.9	62.8
	228S (*218*)	0	0	0	0	0	0
PB2		(*n* = 73)	(*n* = 626)	(*n* = 773)	(*n* = 1472)	(*n* = 28)	(*n* = 35)
	T271A	0.0	0.2	0	0.1	19.7	0
	526R	0.0	3.4	3.5	3.3	3.8	0
	590S	60.3	12.8	3.7	10.3	30.8	37.1
	591K	0.0	1	1.1	1.0	0	2.9
	590S/591K	0.0	0.3	0	0.1	0	0
	**A588V**	6.9	5	29.1	17.8	46.2	11.4
	E627K	4.1	1	1	1.1	3.6	8.6
	E627V	0.0	12	4.4	7.4	21.4	0
	D701N	0.0	0	0.1	0.1	3.7	2.9
	K702R	26.0	12	2.1	7.5	15.4	42.9
PB1		(*n* = 71)	(*n* = 572)	(*n* = 564)	(*n* = 1207)	(*n* = 16)	(*n* = 24)
	H99Y	0	0	0	0	0	0
	327K	0.0	0.5	0	0.2	0	0
	336I	5.6	1.2	0.2	1.0	0	0
	**I368V**	2.8	21.3	67.4	41.8	62.5	33.3
PA		(*n* = 46)	(*n* = 605)	(*n* = 394)	(*n* = 1045)	(*n* = 28)	(*n* = 27)
	85I	0.0	0.2	0.0	0.1	0.0	0.0
	86S	0.0	0.0	0.0	0.0	0.0	0.0
	**100A**	0.0	0.7	14.7	5.9	0.0	18.5
	336M	0.0	4.1	4.6	4.1	14.3	0.0
	**356R**	2.0	5.5	82.7	34.4	57.1	25.9
	**409N**	9.8	36.9	82.5	59.3	60.7	66.7

^a^ Mature H3 HA numbering (mature H9 HA numbering). ^b^ Boldface type indicates a residue displaying increased prevalence over time. ^c^ The total number of sequences from GISAID that were analyzed for each group.
